# Westernization of the Asian Nose by Augmentation of the Retropositioned Anterior Nasal Spine With an Injectable Filler

**Published:** 2011-02-16

**Authors:** Yohei Tanaka, Kiyoshi Matsuo, Shunsuke Yuzuriha

**Affiliations:** Department of Plastic and Reconstructive Surgery, Shinshu University School of Medicine, Matsumoto, Nagano, Japan

## Abstract

**Objective:** The aging nose presents as a drooping nasal tip secondary to atrophy of the underlying bony support with a relatively prominent dorsal hump. We hypothesized that low nasal tip projection in the Asian nose may be secondary to the retropositioned anterior nasal spine (ANS) as well. Therefore, we investigated how filler injection between the medial crura and the retropositioned ANS affects the nasal shape. **Methods:** Local anesthetic was injected on the ANS for analgesia and simulation for the following augmentation. To augment the retropositioned ANS, approximately 0.3 to 0.5 mL of injectable hyaluronic acid was injected between the footplates of the medial crura and the ANS. We evaluated 30 patients (29 women and 1 man) ranging from 21 to 71 years of age (36.5 ± 7.2 years) before and after the augmentation. **Results:** Augmentation of the retropositioned ANS significantly decreased nasal width, but increased alar length, nasal tip protrusion, inclination of the nostril axis from the horizontal, and columellolabial angle (*P* < .0001). **Conclusion:** Our findings indicate that augmentation of the ANS elongates the pseudocolumella, lifts the medial crura, and affects the whole nasal shape. Consequently, augmentation of the retropositioned ANS with an injectable filler westernized the Asian nose. This temporary method may be useful to predict the results of ANS augmentation with other permanent fillers and to make a space to be filled with them.

With aging, there is a relative shortening of the lower one-third of the face, which results in a concomitant lengthening of the upper and middle two-thirds, including a relative lengthening of the nose.^1^ On the contrary, the aging nose presents as a drooping nasal tip secondary to atrophy of the underlying bony support with a relatively prominent dorsal hump.^2^,^3^ We hypothesized that low nasal tip projection in the Asian nose may be secondary to the retropositioned anterior nasal spine (ANS) as well. To verify this hypothesis, we confirmed how filler injection between the medial crura and the retropositioned ANS affects the nasal shape.

## MATERIALS AND METHODS

**Figure 1:**
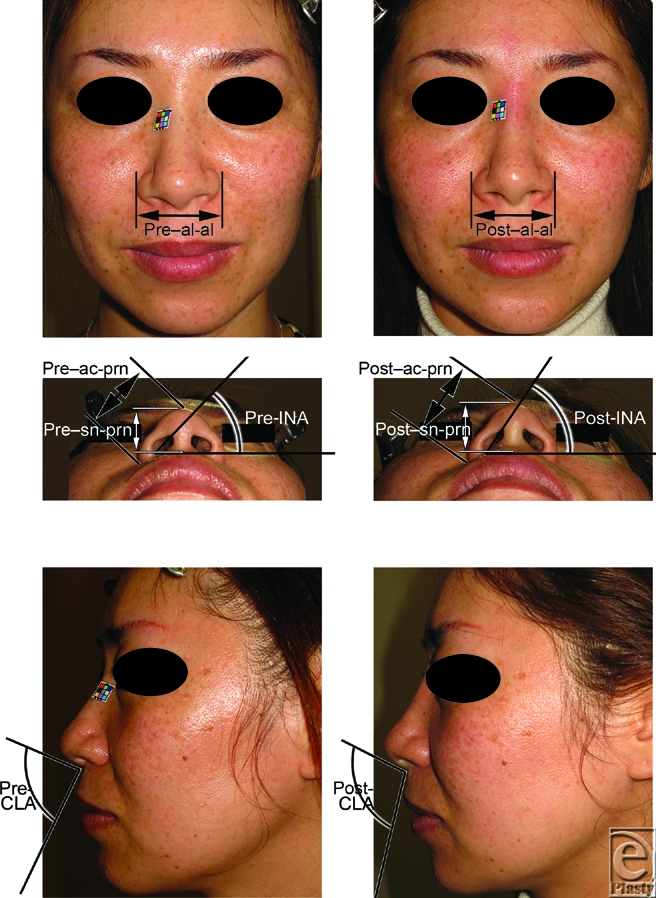
Photographs of the flat lower one-third nose type in a 37-year-old woman. To raise the medial crura, approximately 0.4 mL filler was injected between the medial crura and anterior nasal spine. The injection raised the medial crura, and thus advancement of the lower one third of the nose was achieved, making the flat type of nose into the raised type. (*Above*) The pre- and post-injectional frontal views show a decrease in the morphological nasal width between the alares (al-al). (*Center*) The pre- and post-injectional basal views show increases in the alar length (ac-prn), the nasal tip protrusion (sn-prn), and the inclination of the nostril axis from the horizontal (INA). (*Below*) The pre- and post-injectional side views show an increase in the columellolabial angle (CLA).

**Figure 2:**
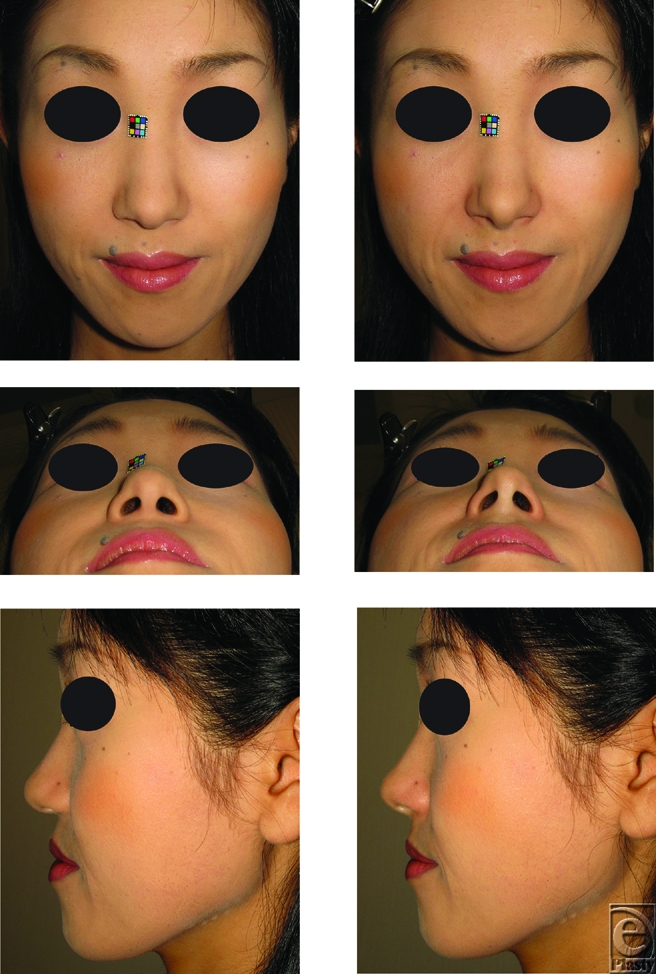
Photographs of the flat lower one-third nose type in a 33-year-old woman. To raise the medial crura, approximately 0.3 mL filler was injected between the medial crura and anterior nasal spine. The injection raised the medial crura, and thus advancement of the lower one-third of the nose was achieved, making the flat type of nose into the raised type. (*Above*) The pre- and post-injectional frontal views. (*Center*) The pre- and post-injectional basal views. (*Below*) The pre- and post-injectional side views.

**Figure 3:**
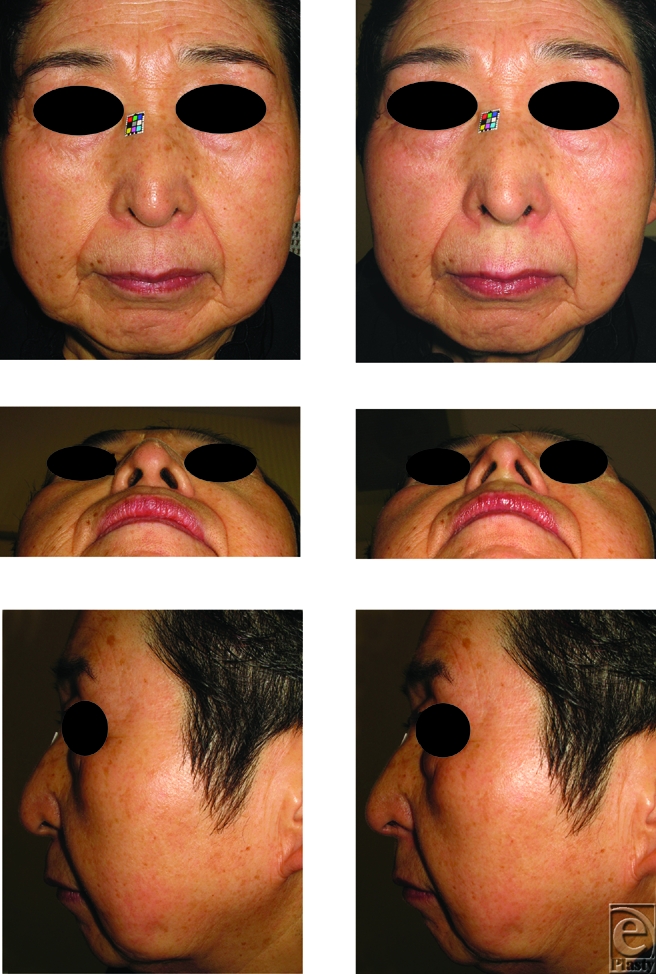
Photographs of the depressed lower one-third nose type in a 71-year-old woman. To raise the medial crura, approximately 0.4 mL filler was injected between the medial crura and anterior nasal spine. The injection raised the medial crura, and thus advancement of the lower one third of the nose was achieved making the depressed type of nose into the flat type. To date, there has not been any sign of airway obstruction. (*Above*) The pre- and post-injectional frontal views. (*Center*) The pre- and post-injectional basal views. (*Below*) The pre- and post-injectional side views.

Local anesthetic was injected to the columellar base for analgesia and simulation for the following augmentation. To augment retropositioned ANS, approximately 0.3-0.5 mL of filler, injectable hyaluronic acid, Restylane (Q-Med, Uppsala, Sweden; and Medicis, Scottsdale, Ariz) was injected between the footplates of the medial crura and the retropositioned ANS in 30 patients (29 women and 1 man) ranging from 21 to 71 years of age (36.5 ± 7.2 years) (Figs 1-3).

As landmarks to evaluate the nasal shape, we chose the alare (al; Fig 1, *above*), which is the most lateral point on each alar contour; alar crest point (ac; Fig 1, *center*), which is the most lateral point in the curved base line of each alar; subnasale (sn; Fig 1, *cente*r), which is the midpoint of the angle at the columellar base where the lower border of the nasal septum and the surface of the upper lip meet; and pronasale (prn; Fig 1, *center*), which is the most protruding point of the apex nasi.

To evaluate the effects of this procedure, the morphological nasal width (al-al; Fig 1, *above*), alar length (ac-prn; Fig 1, *center*), and nasal tip protrusion (sn-prn; Fig 1, *center*) were measured before injection and 7 days after injection, according to the physical anthropology method described by Farkas.^4^ Inclination of the nostril axis from the horizontal (INA; Fig 1, *center*) and columellolabial angle (CLA; Fig 1, *below*) were also calculated.

Data were presented as means ± SD. Comparisons between groups at each time point were performed using paired *t*-test. Differences were considered significant when *P* < .05.

## RESULTS

**Figure 4:**
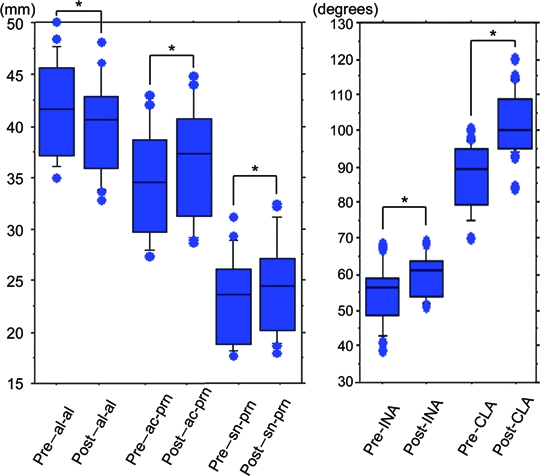
Mean scores pre- and post-injection. There were significant differences between mean pre- and post-injection scores for al-al, ac-prn, sn-prn, INA, and CLA (*P< .0001).

The nasal tip projection was advanced using the procedure described above (Figs 1, 2, and 3). The mean pre-injection scores for al-al, ac-prn, sn-prn, INA, and CLA were 4.162 ± 0.452, 3.449 ± 0.502, 2.315 ± 0.404, 54.62 ± 8.228, and 87.75 ± 8.938, respectively. The mean post-injection scores were 3.972 ± 0.465, 3.655 ± 0.531, 2.427 ± 0.429, 59.7 ± 5.928, and 102.4 ± 9.005, respectively (Fig 4). Augmentation of the retropositioned ANS significantly decreased nasal width (al-al), but increased alar length (ac-prn), nasal tip protrusion (sn-prn), inclination of the nostril axis from the horizontal (INA), and columellolabial angle (CLA) (*P* < .0001).

To date, there have not been any apparent complications such as traction pain, infection, nodules, or skin necrosis.

## DISCUSSION

Augmentation of the retropositioned ANS elongated the pseudocolumella, lifted the medial crura, and affected the whole nasal shape, resulting in westernization of the Asian nose. Although many authors reported that the injectable fillers are useful to improve the facial appearance,^5^^-^9 nobody reported that nasal width (al-al) is decreased by the ANS augmentation. The nasal floors appeared to be secondarily shifted to elongate the columella, resulting in the decrease in nasal width, like Cronin's method for bilateral cleft lip nose deformity.^10^

Successful rhinoplasty depends on the influence of nasal tip support on nasal tip projection.^11^^-^15 In nasal tip support, the footplates of the medial crura play an important role.^16^ The alar cartilage in Asians has been reported to be thin and weak, especially the medial crura, which is responsible for tip projection.^17^^-^19 So far, nasal tip projection has been obtained by lowering the nasal dorsum, trimming or altering the lower lateral cartilages,^20^,^21^ dividing and everting the lower lateral cartilages,^12^,^22^,^23^ using columellar struts,^24^,^25^ tip grafting,^14^,^15^,^26^^-^28 or replanting medial crura.^13^ However, nasal tip projection appeared to be increased by augmentation of the ANS to elongate the columella.

Bimaxillary dentoalveolar protrusion is a common Asian facial morphology.^29^,^30^ The retropositioned ANS emphasizes the bimaxillary dentoalveolar protrusion. Although anterior segmental osteotomy with orthodontic treatment is a method of treating bimaxillary dentoalveolar protrusion, the procedure would worsen the nasal shape because of leaving the ANS retropositioned. We have reported that a rib cartilage graft on the retropositioned ANS improves this sequela in a case of Binder syndrome.^31^

For facial balance, Ricketts recommended the esthetic plane, a line from the nasal tip to the pogonion, from which the upper and lower lips should fall 4 and 2 mm, respectively.^32^ Although some augmentation rhinoplasties have been used to camouflage dentoalveolar protrusion,^29^ these procedures could neither camouflage the position of the ANS nor improve CLA. Our procedure could disguise the retropositioned ANS and improve CLA to approximate Rickett' esthetic plane by projecting the nasal tip. Our findings indicate that the strut of the medial crura plays an important role in nasal tip projection even in the Asian nose.

In conclusions, the low nasal tip projection in the Asian nose may be secondary to the retropositioned ANS as well as the drooping nasal tip in the Western elders. We could confirm that filler injection between the medial crura and the retropositioned ANS westernizes the Asian nose. This temporary method may be useful to predict the results of ANS augmentation with other permanent fillers and to make a space to be filled with them.
